# Preparation, Binding Behavior and Molecular Simulation of Binary Complexes of Phloridzin with Whey Protein Isolate

**DOI:** 10.3390/foods15122089

**Published:** 2026-06-09

**Authors:** Jiaqi Li, Nanjun Liu, Furong Qin, Chenxi Qiu, Li Fu, Yinchen Hou, Xueqin Gao

**Affiliations:** College of Food Science and Engineering, Henan University of Animal Husbandry and Economy, Zhengzhou 450046, China; 15937322777@163.com (J.L.);

**Keywords:** whey protein isolate, phloridzin, interaction mechanism, molecular dynamics, complex

## Abstract

Whey protein isolate (WPI) can assemble into supramolecular complexes with flavonoids via non-covalent interactions, although the underlying binding mechanisms remain not fully understood. In this work, the formation mechanism of the WPI–phloridzin (PHL) complex was systematically investigated using an integrated experimental and computational approach. High-performance liquid chromatography quantified the binding content of PHL as 1.3% (*w*/*w*). Isothermal titration calorimetry indicated that the process was entropy-driven and governed predominantly by hydrophobic and electrostatic interactions. Complementary circular dichroism spectroscopy and molecular dynamics simulations revealed that complexation induces modest conformational adjustments in the protein’s secondary structure. Collectively, this multi-scale analysis provides mechanistic insights into the dynamic formation of the WPI–PHL complex, offering theoretical insights into protein–flavonoid recognition.

## 1. Introduction

Phloridzin (PHL) is a polyphenolic compound found in a number of different plants, mainly from the families *Rosaceae* and *Ericaceae*. It is the predominant phenolic compound in plants belonging to the *Malus* species, which contain high PHL concentrations in their leaves and bark [1]. Due to its natural occurrence in apples and apple-based products, humans have been chronically exposed to PHL through diet for centuries, with estimated daily intakes as high as 52 mg/day for high-level consumers [2]. The key to the biological activity of polyphenols lies in their stability in the gastrointestinal tract and targeted delivery ability. Polyphenols are more stable under acidic conditions. However, the near-neutral pH environment in the small intestine accelerates their degradation [3]. During gastrointestinal digestion, oxidative degradation of polyphenols is often observed [4]; polyphenols may also be sequestered by forming complexes with digestive enzymes. Proteins have amino, carboxyl and hydroxyl groups, which bind to compounds such as polyphenols. A large number of studies have shown that polyphenols exhibit strong binding affinity with proteins through non-covalent or covalent bonds, thereby changing the structure and properties of proteins, thus significantly affecting the accessibility and bioavailability of flavonoids [5]. Studies have shown that proteins perform well as protective agents and carriers of polyphenols, effectively preventing them from being subjected to enzymatic hydrolysis and oxidative degradation during gastrointestinal digestion, and safely delivering them to the small intestine and colon [6].

Currently, research on binary complexes of flavonoids with whey protein isolate (WPI) has primarily focused on complex preparation and characterization, while systematic investigations into their interaction mechanisms at the atomic level remain limited, although molecular dynamics (MD) simulations have been widely employed to study protein–polyphenol interactions [7]. Most existing studies have concentrated on well-known flavonoids such as quercetin or resveratrol in complex with WPI or its major components [8,9]. In contrast, the molecular interaction mechanism between PHL—a dihydrochalcone with distinct structural features—and WPI has received relatively limited attention, particularly regarding systematic elucidation using MD simulations coupled with visualization techniques.

Molecular simulation methods employ theoretical calculations to simulate intermolecular interaction processes. Among these, MD simulation analyzes the structural and functional properties of molecules by studying atomic motions and interatomic interactions [10], providing valuable information about molecular interaction systems and enabling observation of protein structural changes. The independent gradient model (IGM) is a recently developed visualization approach that enables the identification and graphical representation of non-covalent interactions, such as hydrogen bonds, van der Waals forces, and steric effects, based on electron density gradients [11]. Unlike conventional MD simulations, which primarily provide quantitative binding energies and dynamic structural information, IGM offers intuitive visualization of interaction regions and enables discrimination between different types of intermolecular forces. The key advantage of combining MD with IGM analysis lies in its ability to not only quantify binding energies but also to visualize the spatial distribution and types of interactions at the atomic level, providing a more comprehensive understanding of the recognition mechanism between WPI and PHL.

Based on this, we adopt an integrated strategy of combining experimental and computational methods to elucidate the microscopic binding behavior between WPI and PHL. Unlike previous studies that primarily focused on binding energy calculations and structural analysis using MD simulations alone, the effects of PHL on protein structure were comprehensively characterized using circular dichroism spectroscopy and molecular dynamics simulation. Binding energies and interaction forces within the supramolecular complex were investigated, and the IGM was employed to visualize intermolecular interactions—enabling the identification of interaction types and their spatial distribution, which cannot be obtained from MD simulations alone. This combined approach offers a more comprehensive perspective than conventional MD-based studies, enabling a more systematic and in-depth exploration of the interaction mechanism between WPI and PHL.

The findings of this study provide methodological support for the study of intermolecular interactions and have significant implications for broadening the application of PHL in the functional food industry.

## 2. Materials and Methods

### 2.1. Materials

WPI (93.77% protein content, dry basis) was purchased from Mullins Whey Inc. (Mosinee, WI, USA) and was purified from cow milk. α-Lactalbumin (α-La, purity ≥ 85% by SDS-PAGE, from bovine milk, product L5385) and β-Lactoglobulin (β-Lg, purity ≥ 90% by SDS-PAGE, from bovine milk, product L3908) were purchased from Sigma-Aldrich (St. Louis, MO, USA). Phloridzin (PHL, 98% purity, anhydrous, CAS 60-81-1) was obtained from Xi’an Henderson Chemical Co., Ltd. (Xi’an, China). Ultrapure water (resistivity ≥ 18.2 MΩ·cm, conductivity ≤ 0.055 µS/cm at 25 °C) was obtained from a Thermo Gen Pure ultraviolet (UV)/ultrafiltration water system (Waltham, MA, USA). Analytical weighing was performed using an ME104 analytical balance (Mettler-Toledo GmbH, Greifensee, Switzerland; readability: 0.1 mg).

### 2.2. Preparation of Complexes

WPI (450 mg) and PHL (100 mg) were mixed and dissolved in 100 mL of deionized water; 10 mg of sodium azide was added to prevent microbial growth. The mixture was held at 30 °C with shaking for 48 h using a SHA-C water bath thermostatic oscillator (Dazhong Instrument Factory, Jintan, Jiangsu, China) operated in horizontal reciprocating mode with a shaking orbit of 20 mm (amplitude) at a shaking frequency of 150 rpm [12]. After incubation, the mixture was centrifuged at 10,000× *g* for 20 min at 4 °C. The supernatant was then dialyzed using a dialysis bag (22 mm flat width, molecular weight cut-off 3500 Da) against 500 mL of deionized water at 4 °C for 72 h; the outer buffer was replaced every 12 h. The dialyzed samples were collected by centrifugation, and the supernatant was taken and freeze-dried using an Alpha 1–2 LD freeze dryer (Marin Christ GmbH, Osterode, Germany). The sample was pre-frozen at −80 °C for 12 h. Lyophilization was carried out with a condenser temperature of ≤−55 °C and a chamber pressure of ≤2 × 10^−3^ mbar for 48 h. The resulting freeze-dried product is referred to as the WPI–PHL binary mixture.

### 2.3. High-Performance Liquid Chromatography

Standard curve: The external standard method was used to establish the standard curve of PHL concentration and peak area. The standard solution of PHL was 0.1 mg·mL^−1^ (anhydrous basis), and 0.2, 0.6, 1.0, 1.4, and 1.8 mL of the above standard solution were transferred to a test tube. The solution was diluted with deionized water to 10 mL and then filtered through a 0.45 μm aqueous filter membrane. Finally, the peak area of the sample was determined by an Agilent 1260 high-performance liquid chromatograph (HPLC) (Agilent Technologies, Inc., Santa Clara, CA, USA) equipped with a Variable Wavelength Detector (VWD, flow cell optical path length: 10 mm) and an Agilent ZORBAX SB-C18 column (150 mm × 4.6 mm, 5 μm). The detection wavelength was 290 nm. The mobile phase was methanol and water (65:35, *v*/*v*) at a flow rate of 1 mL·min^−1^, and the injection volume was 20 μL. (Note: When mixing 65 mL methanol and 35 mL water at 25 °C, the total volume is not 100 mL but approximately 96.4 mL due to volume contraction [13]; the mixed solvent is used as the mobile phase without further volume adjustment.) The column temperature was 25 ± 1 °C (room temperature), and the total analysis time per run was 15 min.

For complex analysis, 50 mg of WPI–PHL complex was accurately weighed and diluted with deionized water to a final concentration of 0.1 mg·mL^−1^. The mobile phase conditions were the same as those used for the standard curve. The peak area of the sample was determined. Based on the standard curve, the content of PHL in the complex was calculated.

### 2.4. Circular Dichroism Spectroscopy

Circular dichroism spectroscopy can detect secondary structure changes in proteins. A Chirascan circular dichroism spectrometer (Applied Photophysics Ltd., Leatherhead, UK) with quartz cuvettes of 0.1 cm path length was used to measure the CD spectra of WPI and the WPI–PHL complex. The temperature was 30 °C, the wavelength range was 195–260 nm, the optical path length was 0.1 cm, and the scanning speed was 30 nm min ^−1^. The instrument was calibrated prior to use with a 0.06% (*w*/*v*) solution of ammonium 10-camphorsulfonate (ACS) in ultrapure water, following the manufacturer’s multiwavelength calibration protocol. The circular dichroism spectra of the samples were analyzed by CDtool software [14] to obtain the secondary structure information of the proteins. To correct for the background CD signal originating from PHL itself (which is CD-active due to its glucose moiety [15]), a CD spectrum of PHL (0.1 mg·mL^−1^ in PBS buffer) was determined under identical conditions. This spectrum was then subtracted from the CD spectrum of the WPI–PHL complex using CDtool software (version 1.0), yielding the net CD signal corresponding to protein secondary structure changes.

### 2.5. Isothermal Titration Calorimetry

A Nano-ITC isothermal titration calorimeter (TA Instruments, New Castle, DE, USA) was used to study the interactions of PHL with α-La and β-Lg. PHL was dissolved in PBS (50 mmol·L^−1^, pH 6.8) to obtain a 1 mmol·L^−1^ stock solution, while α-La and β-Lg were each dissolved in PBS (50 mmol·L^−1^, pH 6.8) to final concentrations of 12 μmol·L^−1^. All solutions were degassed under reduced pressure (approx. 67 kPa) for 15 min before ITC analysis. All ITC measurements were carried out at a constant temperature of 25 °C (298.15 K). For each experiment, 1.2 mL of protein solution (α-La or β-Lg, 12 μmol·L^−1^) was placed in the sample cell, and 250 μL of PHL solution (1 mmol·L^−1^) was loaded into the injection syringe. The titration consisted of 30 consecutive injections of 8 μL each at equal intervals. The interactions of PHL with α-La and β-Lg were measured separately. The experimental data were processed using TA Nanoanalyze 3.3 software. The binding parameters were obtained by fitting the data to an independent (one-site) binding model. All ITC experiments were conducted in triplicate (*n* = 3) with freshly prepared protein and ligand solutions to ensure reproducibility.

### 2.6. Molecular Docking

The 3D structure of PHL was downloaded from the PubChem database (https://pubchem.ncbi.nlm.nih.gov/) and optimized using the MOPAC 2016 PM3 method [16] with a gradient norm (GNORM) convergence criterion of 0.01 kcal·mol^−1^·Å^−1^ and the PRECISE keyword to tighten SCF convergence criteria [17]. The crystal structures of α-La (PDB ID: 1F6S) and β-Lg (PDB ID: 3NPO) were obtained from the RCSB Protein Data Bank (https://www.rcsb.org). Molecular docking between each protein and PHL was performed using Autodock 4.2 software. The grid size was set as 60 × 60 × 60 points, and the grid spacing was 0.375 Å [18]. The acceptor rigidity was maintained, while the ligand was treated as flexible. The Lamarckian genetic algorithm (LGA) was applied to search for possible binding poses [19].

### 2.7. Molecular Dynamics

The α-La–PHL and β-Lg–PHL complex conformations obtained from the molecular docking in the previous section (selected as the lowest-energy conformations) were subjected to molecular dynamics simulations. The AMBER99SB-ILDN force field was used for the proteins, while the General AMBER Force Field (GAFF) via the Antechamber tool was used for PHL [20]. Water molecules were automatically added by the gmx solvate program to fill the simulation box. A 1:1 binding model was adopted for molecular docking and MD simulations, focusing on the primary high-affinity binding mode—a standard simplification in protein–ligand interaction studies. The whole system was first energy-minimized using the steepest descent method with periodic boundary conditions. Subsequently, three independent 100 ns isothermal-isobaric ensemble (NPT) simulations at 300 K (v-rescale thermostat, τ = 0.1 ps) were applied to equilibrate the system using the leapfrog algorithm. Trajectory data were recorded every 1 ps, which provided information on root mean square deviation (RMSD), root mean square fluctuation (RMSF), and key centroid distance. The binding free energy (ΔG_bind_) of the complexes and their relative stability in aqueous solvent were analyzed using the gmx_mmpbsa script (https://github.com/Valdes-Tresanco-MS/gmx_MMPBSA, accessed on 8 June 2026), which implements the molecular mechanics Poisson–Boltzmann surface area (MM-PBSA) method. The calculations were performed according to the following formula [21]:

$$∆G_{bind}=G_{complex}-\left(G_{protein}{+G}_{ligand}\right)$$

### 2.8. Independent Gradient Modeling (IGM) to Study Protein–PHL Interactions

In order to explore the intramolecular and intermolecular interactions more deeply and to visualize them more clearly, we used the IGM method to further investigate the α-La–PHL complex and β-Lg–PHL complex systems [22]. The IGM method was used to visualize the intramolecular and intermolecular interactions by calculating the electron density gradients of individual atoms of a molecule and mapping them onto a 3D isosurface map. By expressing the weak molecular interactions as a function of δg, the intramolecular interactions (δg_intra_) and intermolecular interactions (δg_inter_) and their relationship can be expressed as:

δg=δgintra+δginter

Based on the molecular dynamics results, the complex conformation from the last frame was selected for further analysis using the Multiwfn 3.8 program [23]. The resulting data were visualized and processed with VMD 1.9.3 software [24]. The isosurface of δg for the complex system was set to 0.004, and the upper and lower values of the color scale were 5.0 and −5.0, respectively.

### 2.9. Statistical Analysis

The results are expressed as the mean ± standard deviation (SD) from at least three independent replicates. Statistical significance (*p* < 0.05) was determined using one-way analysis of variance (ANOVA), followed by Duncan’s post hoc test.

## 3. Results and Discussion

### 3.1. High-Performance Liquid Chromatographic Analysis

**Figure 1:**
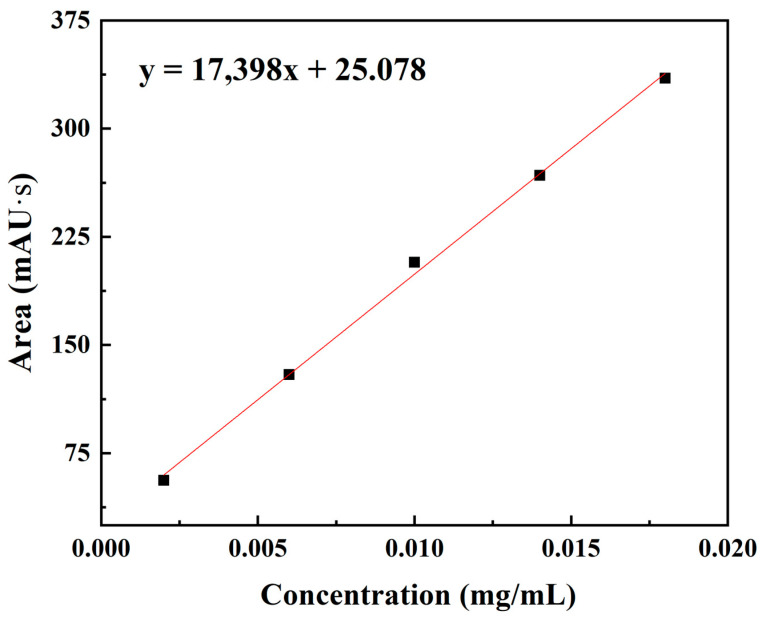
The standard curve of PHL concentration vs. peak area in HPLC.

The standard curve, obtained by plotting the chromatographic peak area against the PHL concentration, showed a good linear relationship (Figure 1). The standard curve was obtained by plotting the peak area (y, in mAU·s) against the PHL concentration (x, in mg·mL^−1^), yielding the linear equation y = 17,398x + 25.078. The peak area of the complex sample (1 mg mL^−1^) was 251 ± 3.03, and the concentration of PHL in the WPI–PHL complex obtained from the PHL standard curve was 0.013 ± 0.0012 mg mL^−1^. That is, the mass percentage of PHL in the lyophilized WPI–PHL complex was 1.3 ± 0.12% (calculated as [PHL concentration]/[complex concentration] × 100%, where the complex concentration was 1 mg mL^−1^). It should be noted that this value represents the amount of PHL that remained associated with WPI after extensive dialysis (4 °C, 72 h, with frequent buffer exchange), which was intentionally performed to remove excess PHL. Therefore, the 1.3% content primarily indicates that the dialysis step was effective in eliminating non-associated PHL, rather than indicating a low binding capacity.

### 3.2. Analysis of Circular Dichroism Spectral Results

**Figure 2:**
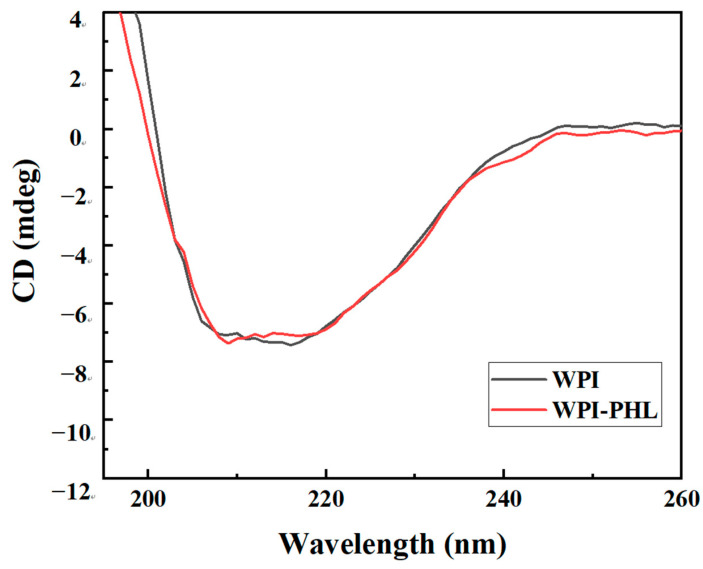
CD spectra of WPI with or without PHL.

The circular dichroism spectra of WPI and WPI–PHL complexes are shown in Figure 2. The presence of a negative broad peak at 210–220 nm for the WPI and complex samples indicates that the proteins in the samples contain a large amount of β-sheet, which is consistent with the literature report that whey protein is a typical β-sheet-rich protein [25].

The secondary structure data of WPI and WPI–PHL complexes, calculated based on circular dichroism spectroscopy, are shown in Table 1. It can be observed that the degree of change in the secondary structure of WPI proteins after complexation with PHL is relatively small. The percentages of α-helix and β-sheet decreased modestly (e.g., antiparallel β-sheet from 27.2% to 25.7%), while β-turn and random coil structures showed slight increases (random coil from 33.3% to 34.56%). This limited decrease in ordered structures and the small increase in less-ordered components suggest that the protein undergoes minor conformational adjustments upon PHL binding. These adjustments may facilitate better access of the small molecule to hydrophobic regions without inducing a major structural rearrangement. Importantly, the overall secondary structure skeleton of the proteins remains largely unchanged, indicating that complex formation does not significantly disrupt the protein fold. We acknowledge that CD normalization for heterogeneous WPI is challenging; therefore, the reported values should be interpreted as estimates focusing on relative changes upon PHL binding rather than absolute structural contents.

### 3.3. Analysis of Isothermal Titration Calorimetry Results

With the improvement of calorimeter sensitivity and accuracy, isothermal titration calorimetry has become an important method for measuring thermodynamic changes during the interaction of various compounds [26,27]. In addition, since ITC assays are based solely on the heat exchange during the reaction, they can detect reaction processes that are independent of spectral changes. Whey protein isolate (WPI) consists of β-lactoglobulin (67.6–74.8%), α-lactalbumin (8.3–17.5%), bovine serum albumin (7.2–10.9%) and immunoglobulins (5.9–7.5%) [28]. To better understand the interactions of different WPI proteins in WPI with PHL, we chose to study the binding of α-La and β-Lg with PHL separately. The thermodynamic parameters obtained from these interactions are shown in Table 2. All ITC experiments were conducted in triplicate, and the thermodynamic parameters in Table 2 are expressed as mean ± standard deviation (SD) calculated from these three independent measurements. According to the ITC-derived binding constants (Ka), the Gibbs free energy changes (ΔG) for the β-Lg/PHL and α-La/PHL complexes were calculated as –22.6 kJ·mol^−1^ and –23.4 kJ·mol^−1^, respectively, using the equation ΔG = –RT ln Ka. These negative ΔG values confirm that both binding processes are spontaneous under the experimental conditions (25 °C). The stoichiometry values (N) obtained from fitting the ITC data to a one-site binding model were 1.7 ± 0.2 for β-Lg/PHL and 1.5 ± 0.4 for α-La/PHL. These values indicate that, under the experimental conditions, each protein molecule binds on average more than one PHL molecule. The non-integer values may reflect a mixture of binding events or partial site occupancy; however, the data quality did not support fitting to a more complex model (e.g., two-site model) without risking overfitting. Given that the primary binding site is of most interest for understanding the molecular recognition mechanism, subsequent molecular simulations were performed using a 1:1 binding model focusing on the highest-affinity interaction.

**Table 2:** The binding parameters of PHL with α-La and β-Lg at 25 °C.

	Ka (×10^3^ M^−1^)	K_d_ (μM)	ΔH (kJ·mol^−1^)	ΔS (J·mol^−1^K^−1^)	N
β-Lactoglobulin/PHL	9.0 ± 0.7 ^a^	111 ± 15	−10.8 ± 1.6 ^c^	58.6 ± 5.5 ^e^	1.7 ± 0.2 ^f^
α-Lactalbumin/PHL	12.3 ± 2.5 ^b^	81 ± 34	−12.7 ± 1.9 ^d^	54.6 ± 7.2 ^e^	1.5 ± 0.4 ^f^

Values are given as the mean ± SD; ^a–f^ means in the same column with different letters differ significantly (*p* < 0.05).

**Figure 3:**
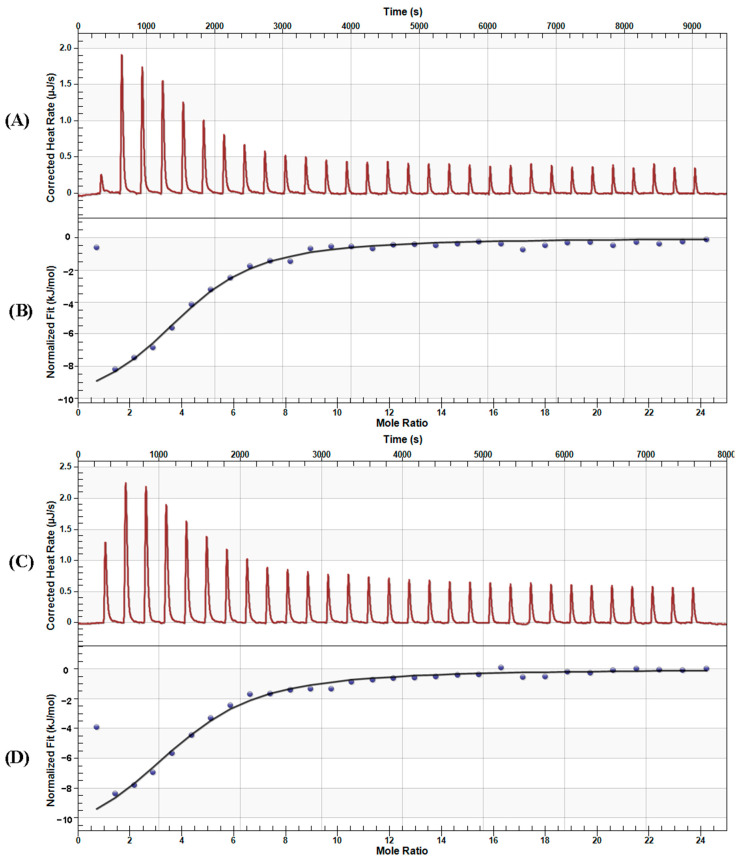
Isothermal titration calorimetry profile for the binding of protein to PHL (**A**,**C**); Plot of heat evolved (kJ) per mole of PHL added against the molar ratio of PHL to protein (**B**,**D**). (The data shown are representative of three independent experiments performed in triplicate (*n* = 3). The dose–response curves integrate the heat change per injection as a function of the [PHL]/[protein] molar ratio).

Figure 3 shows the calorimetric titration curves of PHL with α-La (A, B) and β-Lg (C, D). The peaks in Figure 3A,C represent the heat detected upon sequential injection of the PHL solution into the protein cell. Figure 3B,D show the heat released per injection plotted against the molar ratio of PHL to protein. The fitted curves calculated by the software agree well with the experimental data. Because the ionization enthalpy of phosphate is small [29], the measured enthalpy directly reflects the enthalpy of the interaction between the protein and PHL.

Observation of the peak patterns produced by the titration of PHL with the two proteins in the figure reveals that the binding of PHL with the two proteins is an exothermic process (ΔH < 0). The entropy changes (ΔS) are positive (β-Lg: +58.6 J·mol^−1^·K^−1^; α-La: +54.6 J·mol^−1^·K^−1^), indicating that the binding is accompanied by a decrease in enthalpy and an increase in entropy. For interactions driven primarily by hydrophobic forces, both ΔH and ΔS are typically positive. The combination of negative ΔH and positive ΔS observed here suggests that van der Waals forces and hydrogen bonding also contribute significantly to the binding process, in addition to hydrophobic interactions. The thermodynamic signatures of polyphenol–protein binding can vary depending on the specific ligand structure and binding mode. For example, Ma et al. (2021) reported that the binding of epigallocatechin (EGC) to α-lactalbumin was enthalpy-driven with a negative ΔS (−249 J·mol^−1^·K^−1^), in contrast to our observation of a positive ΔS for PHL binding [30]. This difference likely reflects the distinct chemical structures of the two polyphenols (EGC is a flavan-3-ol with a galloyl group, whereas PHL is a dihydrochalcone glucoside) and their different binding sites on the protein (EGC binds to an exterior surface site, while PHL predominantly occupies the hydrophobic calyx/cleft regions). Both thermodynamic behaviors are physically plausible and consistent with the well-known phenomenon of enthalpy–entropy compensation in protein–ligand interactions [31].

It should be noted that the ITC measurements in this study were performed with isolated β-Lg and α-La, rather than with intact WPI. While these two proteins represent the major protein fraction of WPI, commercial WPI also contains minor amounts of other proteins, including bovine serum albumin, immunoglobulins, and glycomacropeptide [32], which may also bind to PHL and contribute to the overall ITC signal of the complete mixture. Therefore, the thermodynamic parameters obtained in this study primarily reflect the binding properties of the two predominant proteins, and the potential contributions from minor components remain to be investigated in future work.

### 3.4. Molecular Dynamics Simulation Results

**Figure 4:**
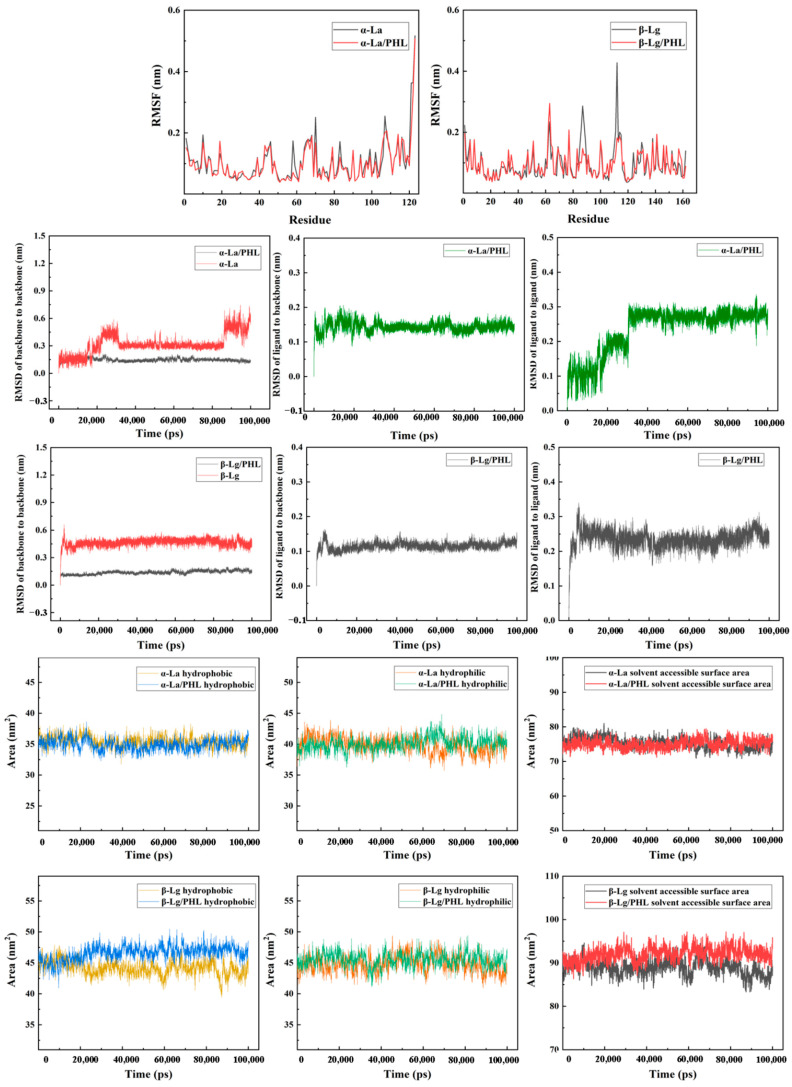
RMSF, RMSD results and solvent-accessible surface area (SASA) of α-La/PHL and β-Lg/PHL in the molecular dynamics simulations.

The initial conformations of the α-La–PHL and β-Lg–PHL complexes obtained from molecular docking (see Table S1 in the Supplementary Materials for the docking results) were subjected to 100 ns molecular dynamics simulations. The resulting RMSD trajectories are shown in Figure 4. The systems reached dynamic equilibrium after approximately 60 ns, indicating that the subsequent simulation data are suitable for interaction analysis.

The RMSF results at 40 ns of the molecular dynamics simulation (Figure 4) reflect the fluctuations of protein residues, which can be observed in the state of amino acid residues in the proteins with or without the presence of PHL molecules. Most amino acid residues of both proteins became more stable and less fluctuating after PHL binding, indicating that the MD simulation of the complexes is in equilibrium. Consistent with the molecular docking results, residues Glu49, Tyr103, Trp104 and Lys108 in α-La exhibited larger fluctuations. These residues are located in the hydrophobic binding pockets, suggesting that their greater mobility may be related to their active participation in molecular interactions with PHL. In contrast, residues Ile84, Phe105 and Val118 in β-Lg showed greater fluctuations than the other amino acids. These residues are located in the water transport pocket of β-Lg, suggesting that they also play an important role in the interactions with PHL, as revealed by the RMSF analysis.

The SASA reflects changes in the hydrophilic/hydrophobic properties of proteins, which in turn affect protein conformation and small-molecule binding ability. The SASA of α-La and β-Lg in the absence and presence of PHL over the 100 ns MD simulation are shown in Figure 4. The presence of PHL increased the hydrophobic SASA of β-Lg to a greater extent than that of α-La, while its effect on hydrophilicity was smaller. This suggests that during PHL binding, β-Lg undergoes a greater degree of structural loosening, with more hydrophobic amino acids exposed to the solvent, leading to an increase in SASA.

The average SASA values of the protein–PHL system over the last 40 ns of the MD simulations were calculated to investigate protein structural changes (Table 3). Upon PHL binding, the SASA of both proteins increased. For α-La, the average SASA over the last 40 ns increased by 1.04 nm^2^, compared to the free protein, whereas for β-Lg, the increase was 3.95 nm^2^. Thus, the binding of PHL had a greater effect on the molecular structure of β-Lg.

**Figure 5:**
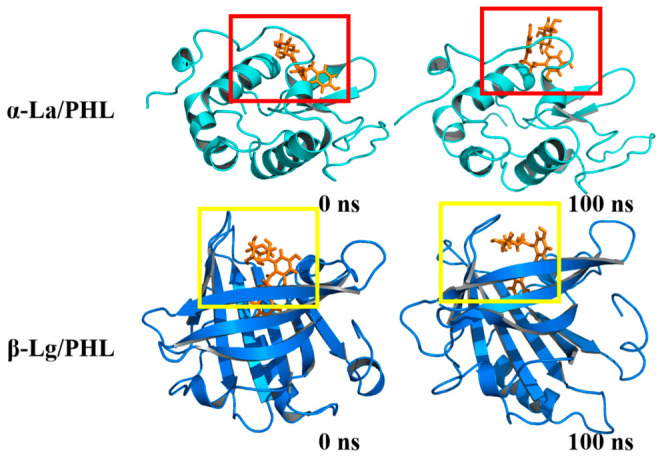
The conformations of α-La–PHL and β-Lg–PHL complexes at 0 and 100 ns (the water molecule model is hidden).

Comparison of the initial and final conformations from the MD simulations (shown in Figure 5) is also consistent with the above results. The overall protein backbone remains essentially unchanged, and the binding site is relatively fixed, whereas the conformation of the PHL molecule within the hydrophobic pocket (see Figure S1 in the Supplementary Materials for the chemical structure of PHL) is somewhat twisted. This indicates that the protein backbone remains a nearly rigid structure during the simulation, while the PHL molecules in the binding site exhibit greater flexibility, allowing them to adapt their conformation for tighter binding to the protein.

The calculated average free energy data of the complexes, based on the last 40 ns trajectory of the system MD simulation, are shown in Table 4. The binding free energies in both systems are negative, indicating that the binding process can proceed spontaneously. The binding free energy of the PHL with α-La (−485.0 ± 0.8 kJ/mol) was more negative than that with β-Lg (−77.5 ± 0.5 kJ/mol), indicating that PHL has stronger affinity for α-La than for β-Lg. This is consistent with the trends observed in molecular docking and ITC experiments. Electrostatic interactions and SASA (non-polar solvation) energy play important roles in the binding process, whereas polar solvation energy partially counteracts these attractive contributions.

### 3.5. Analysis of IGM Intermolecular Interactions

**Figure 6:**
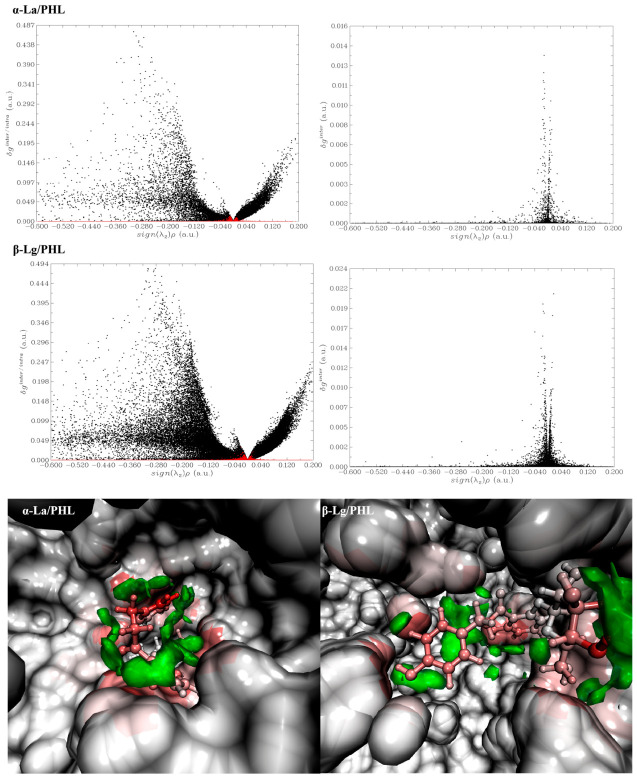
The scatter plots of δg^inter/intra^ vs. sign(λ_2_)ρ (the red and black points correspond to δg^inter^ and δg^intra^, respectively) and the visualization of intermolecular interactions between the protein and PHL (green isosurface indicates the interaction region). (Here, sign(λ_2_) is defined as +1 for λ_2_ > 0, 0 for λ_2_ = 0, and –1 for λ_2_ < 0).

The scatter plots of intramolecular δg_intra_, intermolecular δg_inter_ and sign(λ_2_)ρ calculated by the IGM method are shown in Figure 6, where the red scatter points represent intermolecular interactions and the black scatter points represent intramolecular interactions, and the densely distributed δg_inter_ scatter points near sign(λ_2_)ρ = 0 in the figure indicate that hydrogen bonding and van der Waals forces are present in the complexes. Sign(λ_2_)ρ is defined as the product of the electron density ρ and the sign of the second eigenvalue (λ_2_) of the electron density Hessian matrix; the sign function returns +1 for λ_2_ > 0, 0 for λ_2_ = 0, and −1 for λ_2_ < 0.

The δg_inter_ isosurface maps obtained using VMD and the 3D conformations of the binding sites, colored according to the δg indices of the atoms, are shown in Figure 6. The green areas in the maps are the isosurfaces of the δg_inter_; the green globular areas (e.g., near the hydrogen atoms on the phenol group of PHL) indicate the presence of hydrogen bonding, and the lamellar isosurface areas are the van der Waals forces. Since the color scale of VMD is set to blue-white-red (BWR), larger δg indices of the atoms in the figure show a deeper degree of redness and stronger interactions, whereas white atoms indicate that their δg indices are close to 0, contributing little to the interactions. In Figure 6, the glycosidic and phenolic groups of the PHL molecule and the amino acid residues in its vicinity show a deeper red color, indicating that PHL binds well to both proteins, and that the atoms in the hydrophobic amino acids in the binding region and in the phenolic group of the PHL contribute to the intermolecular interactions to a greater extent. In summary, the IGM analysis identifies the phenolic hydroxyl, glycosidic hydroxyl, and aromatic ring groups of PHL as the key atomic moieties responsible for the observed intermolecular interactions. Thus, the IGM method can clearly visualize the key atoms involved in the interactions in the protein–PHL complex system, which facilitates the investigation of the location and strength of weak intermolecular/intramolecular interactions in the protein–small molecule system.

## 4. Discussion

### 4.1. Interpretation of Binding Interactions in Light of Prior Literature

Our results confirm that phloridzin (PHL) forms a stable supramolecular complex with whey protein isolate (WPI), primarily through non-covalent interactions. This finding aligns with the growing consensus that hydrogen bonding and van der Waals forces dominate protein–polyphenol binding, especially when the polyphenol contains multiple hydroxyl groups and a glucoside moiety [33,34].

(a) Glycoside contribution:

Contrary to earlier assumptions that bulky glucoside groups at C-3 and C-7 hinder binding to enzymes such as xanthine oxidase [35], our QSAR and IGM analyses reveal that the glucoside at C-5 actually enhances the interactions with WPI. This is consistent with Lefebvre’s IGM, which demonstrates that the glycosidic oxygen atoms act as strong hydrogen-bond acceptors, forming additional H-bonds with residues like Asp-101 and Ser-126 in α-lactalbumin (α-La). Recent spectrofluorometric studies also reported that flavonoids with glycosylated sugars exhibit higher binding constants to whey proteins compared with their aglycone counterparts [36].

(b) Protein flexibility:

The MD simulations showed an increase in the solvent-accessible surface area (SASA) of both α-La and β-lg upon PHL binding, indicating that the proteins adopt a more flexible, open conformation. This observation mirrors the “protein-induced fit” model reported for catechin–β-lg complexes, where the protein undergoes conformational rearrangements to accommodate the polyphenol [37]. Such flexibility may facilitate the encapsulation of PHL within the hydrophobic cavities of WPI, thereby protecting it from oxidative degradation—a hypothesis supported by our CD data showing a reduction in ordered secondary structures.

(c) Thermodynamic consistency:

The negative ΔG values derived from ITC, together with the exothermic ΔH and favorable ΔS, suggest that the binding process is both enthalpically and entropically driven. This dual driving force is characteristic of interactions where hydrogen bonding (enthalpic contribution) co-exists with hydrophobic burial of aromatic rings (entropic contribution). The stronger binding affinity observed for α-La (K = 1.23 × 10^4^ M^−1^) compared with β-lg (K = 9.04 × 10^3^ M^−1^) may be attributed to the higher proportion of positively charged residues (e.g., Lys, Arg) on α-La that can interact electrostatically with the negatively charged phenolate forms of PHL at physiological pH [18].

### 4.2. Study Limitations

While the current study provides a comprehensive characterization of the WPI–PHL complex, several limitations should be acknowledged:

(a) In vitro vs. in vivo relevance:

All binding assays (ITC, CD, MD) were performed under controlled laboratory conditions (pH 6.8, 25 °C). The gastrointestinal environment is far more complex, with varying pH, presence of digestive enzymes, and competing ligands that could alter the binding equilibrium. Future work should incorporate simulated digestion models to assess the stability of the complex under physiological conditions.

(b) Protein heterogeneity:

Commercial WPI is a mixture of α-La, β-lg, bovine serum albumin (BSA), and minor proteins. In this study, we investigated α-La and β-lg separately, but the synergistic or competitive interactions among these proteins in the native WPI matrix were not explored. Such interactions could affect the overall encapsulation efficiency and release kinetics of PHL.

(c) Simplified binding stoichiometry:

The ITC data suggested multiple binding sites (N > 1), yet the MD simulations focused on a 1:1 binding model for computational tractability. This simplification may overlook cooperative binding effects or allosteric changes that occur when multiple PHL molecules bind simultaneously to a single protein molecule.

(d) Limited spectroscopic validation:

While CD and HPLC provided valuable insights, additional techniques such as NMR (for atomic-level interaction mapping) or small-angle X-ray scattering (SAXS) for solution-state structural changes could further validate the proposed binding modes.

### 4.3. Future Research Directions

Addressing the aforementioned limitations will be crucial for translating these findings into functional food or nutraceutical applications:

(a) Simulated Digestion and Bioavailability Studies:

Conduct in vitro digestion assays (e.g., INFOGEST protocol) followed by Caco-2 cell transport studies to evaluate whether WPI encapsulation truly enhances the bioaccessibility and intestinal permeability of PHL. Parallel in vivo pharmacokinetic studies in rodent models could quantify improvements in plasma half-life and tissue distribution.

(b) Protein Engineering via AI-Driven Design:

Leverage AlphaFold-Multimer or RoseTTAFold to design mutant whey proteins with increased surface hydrophobicity or additional charged residues at strategic positions. This could potentially boost the binding affinity for PHL, as suggested by recent AI-guided protein–ligand interaction studies [38,39].

(c) Multi-Component System Optimization:

Explore the effect of co-encapsulation of PHL with other bioactives (e.g., vitamins, minerals, or other polyphenols) to assess synergistic antioxidant effects. Additionally, investigate how the presence of dietary fibers or polysaccharides (e.g., pectin, alginate) influences the release profile and sensory attributes of the final product.

(d) Advanced Structural Characterization:

Apply cryogenic electron microscopy (cryo-EM) or solid-state NMR to capture the three-dimensional architecture of the WPI–PHL complex at near-atomic resolution. This could provide definitive evidence for the proposed “encapsulation within hydrophobic pockets” hypothesis.

(e) Safety and Sensory Evaluation:

Perform long-term stability tests under various storage conditions (temperature, humidity, light exposure) and conduct sensory panels to assess any residual bitterness or astringency, ensuring that the protein matrix effectively masks the inherent sensory drawbacks of PHL.

## 5. Conclusions

The results indicated that the ordered structure of the protein experienced a slight reduction during the binding process of phloridzin (PHL) with WPI, accompanied by a modest increase in random coil content (from 33.28% to 34.56%), which may be due to the enhanced structural flexibility of the protein during the interaction, which facilitates the better access of small molecules into the hydrophobic pockets and the formation of complexes. In addition, the binding of PHL to both α-La and β-Lg in WPI is an exothermic process capable of proceeding spontaneously, with the hydroxyl groups of PHL forming hydro-gen bonds with amino acid residues, and the aromatic rings contributing to hydrophobic interactions. The binding free energy and binding constants obtained from ITC and molecular simulations indicate that PHL exhibits a greater (more negative) binding free energy and higher binding constants for α-La than for β-Lg, implying a stronger binding affinity toward α-La. These findings suggest that WPI–PHL complexes have potential as functional food ingredients, where WPI can serve as an effective carrier to improve the stability and bioavailability of PHL. Future studies should evaluate the protective effect of this complex under simulated gastrointestinal conditions and assess its performance in real food matrices, as well as identify the precise locations of secondary binding sites using site-directed mutagenesis.

## Figures and Tables

**Table 1:** Changes in secondary structure of WPI interacting with PHL.

	α-Helix (%)	Antiparallel Structure (%)	Parallel Structure (%)	β-Turn (%)	Random Coil (%)
WPI	14.3 ± 0.2 ^a^	27.2 ± 0.1 ^a^	5.58 ± 0.09 ^a^	19.3 ± 0.2 ^a^	33.3 ± 0.2 ^a^
WPI–PHL	13.3 ± 0.2 ^b^	25.7 ± 0.1 ^b^	5.3 ± 0.1 ^b^	19.8 ± 0.1 ^b^	34.56 ± 0.05 ^b^

Values are given as the mean ± SD; ^a,b^ means in the same column with different letters differ significantly (*p* < 0.05).

**Table 3:** Average solvent-accessible surface area (SASA) in the last 40 ns of MD simulations.

System	SASA (nm^2^)
α-La	74.5 ± 1.4 ^a^
α-La/PHL	75.5 ± 1.4 ^b^
β-Lg	88.6 ± 2.0 ^a^
β-Lg/PHL	92.5 ± 1.5 ^b^

Values are given as the mean ± SD; ^a,b^ means in the same column with different letters differ significantly (*p* < 0.05).

**Table 4:** MM/PBSA average free energy of the complexes calculated from the MD simulations from 4000 frames.

Name	van der Waal Energy (kJ/mol)	Electrostatic Energy (kJ/mol)	Polar Solvation Energy (kJ/mol)	SASA Energy (kJ/mol)	Binding Energy (kJ/mol)
α-La/PHL	74.6 ± 0.5 ^a^	−2123 ± 2 ^a^	1566 ± 2 ^a^	−2.790 ± 0.004 ^a^	−485.0 ± 0.8 ^a^
β-Lg/PHL	−180.7 ± 0.4 ^b^	−48.1 ± 0.8 ^b^	173 ± 1 ^b^	−21.86 ± 0.031 ^b^	−77.5 ± 0.5 ^b^

Values are given as the mean ± SD; ^a,b^ means in the same column with different letters differ significantly (*p* < 0.05).

## Data Availability

The original contributions presented in this study are included in the article/Supplementary Materials. Further inquiries can be directed to the corresponding authors.

## Supplementary Materials

The following supporting information can be downloaded at: https://www.mdpi.com/article/10.3390/foods15122089/s1, Figure S1. Molecular structure of PHL; Table S1. Results of molecular docking.
