# Supplementary material for: Preparation, Binding Behavior and Molecular Simulation of Binary Complexes of Phloridzin with Whey Protein Isolate
Source: Foods. 2026 Jun 9;15(12):2089. doi: 10.3390/foods15122089 (PMC13297784; doi:10.3390/foods15122089)
Supplement: Supplementary file 1 [file foods-15-02089-s001.zip › foods-4195013-supplementary.pdf]

Supplementary Materials

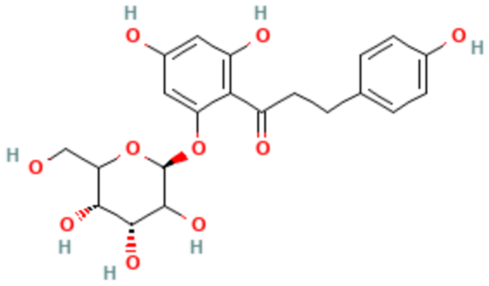

Figure S1. Molecular structure of PHL.

Table S1. Results of molecular docking.

System	Binding Energy (kcal/mol)
β-Lg/PHL	-3.13
α-La/PHL	-4.64
